# Active control of noise radiated from a long cylinder: Can the Nyquist limitation be broken?

**DOI:** 10.1016/j.heliyon.2024.e37670

**Published:** 2024-09-11

**Authors:** N. Hu, S. Utyuzhnikov

**Affiliations:** Department of Fluids and Environment, University of Manchester, Manchester, M13 9PL, UK

**Keywords:** Active noise control, Noise attenuation, Surface potentials, Green's function, Nyquist-Shannon sampling theorem

## Abstract

In the present paper, we consider active control of noise propagating from a long cylinder. For that purpose control sources are distributed on the external surface of the cylinder. They provide a secondary sound field which enables them to compensate for the noise field propagating outside. To implement active noise control, we apply an approach based on the Calderón potentials that have the projection property. Numerical simulations to attenuate broadband noise with different numbers of control sources per wavelength are carried out. The effect of the cylinder on the Green's function and consequent noise attenuation is studied. There are two key findings in this paper. First, the results demonstrate that the level of relative noise attenuation remarkably increases along with the growth of the distance. Second, it is shown that even less than two control sources per wavelength can provide essential noise cancelation. At first glance, this result contradicts the well-known Nyquist-Shannon sampling theorem. A theoretical explanation of this result is provided.

## 1 Introduction

Power plants can produce significant noise through steam ducts such as an air-cooling condenser. Noise emitted by chemical and power-generating plants can cause a series of problems for neighboring residents. Many people who live near industrial plants are annoyed by the noise and have psychological distress. Therefore, it is important to overcome the noise hazard generated by industrial pipework.

Passive noise control techniques are often applied to such pipes in industry. For example, a soundproofing warp made up of dense vinyl material is implemented for absorbing noise generated by pipes. With passive noise control, mid- and high-frequency noise is partially reduced while noise attenuation in the range of low frequencies is insufficient. It is well known that the active noise control (ANC) is efficient at attenuating low-frequency noise. ANC based on the Huygens principle [1] can volumetrically cancel the noise outside the pipe by generating antinoise with control sources at the boundary. Noise and antinoise waves have the same amplitude but the opposite phases. They neutralize each other to achieve the goal of noise attenuation.

The first concept about ANC was proposed by Lueg in his patent [21], where a monopole is allocated in an unbounded duct to generate antinoise that compensates the incoming noise. The idea of ANC was further developed in the JMC method by Jessel, Mangiante [12], [22] and Canevet [3]. They showed that monopoles and dipoles should be set in pairs to reach noise attenuation in an acoustically shielded region while having no effect outside. In the subsequent development of ANC technique, some approaches require a detailed description of the noise sources (see [13]) in order to realize a global noise attenuation. In practice, however, the information such as the radiation patterns [10], about the noise sources is usually unknown. In such cases, significant noise attenuation can often be attained only locally [14], [24].

There are a number of techniques developed in [3], [8], [9], [12], [14], [24], [27], [29], etc., that require only the measurement of noise at the perimeter of the domain to be protected. Basically, two primary systems of the active noise control exist: feedforward and feedback architectures. The feedforward systems obtain the reference signal before it reaches noise control region which makes the broadband noise control possible [24]. The feedback systems detect the noise at the noise control region which is mostly suitable for narrow band noise control. Both kinds of systems exploit adaptive filters based on the least mean square (LMS) algorithm, whose convergence deteriorates with low frequencies. It should be noted that the LMS algorithm provides noise attenuation near selected points instead of a continuous space. In [15], the individual kernel interpolation of sound fields was proposed to overcome this drawback in the LMS algorithm. In [16], it was shown that the placement method for both sensors and control sources can significantly increase the level of ANC. In addition, Patel et al. [27] modified the FxLMS algorithm to make it more robust to uncertainties.

In application to attenuation of noise propagating from a pipe, various ANC strategies have been proposed under different conditions. For the cancelation of harmonic noise, the active structural acoustic control method is developed in [23]. In this method, discrete force actuators installed on the surface effectively compensate for the vibration of the pipe. The noise generated by the vibrating structure is minimized by optimizing the quadratic expression of the total sound power. For attenuating non-harmonic noise, the direct velocity feedback control method [7] can be employed. This strategy achieves noise attenuation by implementing discrete surface-bonded piezoelectric patches as sensors or control sources on the surface of the pipe. It turns out to be effective even if noise is generated by random excitation. However, the effect of noise attenuation collapses when the nonlinearities of the noise vibration increase. This ANC approach with spatially discrete piezoelectric actuators can be applied to double-wall cylindrical shells as well [38]. In this case, both the velocity feedback and sound pressure rate feedback are taken into consideration. In addition, in [4], the technique for the piezoelectric laminated cylindrical shells is extended to the wavenumber domain. The obtained numerical results show that the size of piezoelectric patches plays an important role in the effect of noise attenuation. Moreover, some other situations involving noise radiated from a pipe are investigated in [4]. For example, the active control of noise radiated from a finite pipe is also investigated for the cases when the pipe is submerged in liquid [6] and filled with liquid [34], respectively. Most of these ANC techniques realize noise attenuation by exerting forces on the cylindrical surface to diminish the vibration of the pipe. However, the vibration of the pipe is not the only factor that generates noise. Gases and liquids flowing through the pipe also emit noise. In this case, the effect of noise reduction mentioned above is undermined. To tackle this problem, an entirely different ANC strategy can be introduced.

In [11], it is demonstrated that the Calderón potentials [2] can be used for a wide range of partial differential equations and have the property of projection. As first noted in [30], the Calderón potentials can effectively be used for ANC. In [30], this methodology is exploited to solve linear stationary ANC problems. A desired sound generated inside the protected region is allowed to be present. It is supposed not to be affected by ANC. A discrete formulation assigned to a discrete space is provided based on the formalism of the method of difference potentials (DPM) [30]. Afterwards, the links between the Calderón potentials in the discrete and continuous spaces are addressed in [20], [35]. This approach is extended to unsteady problems including the acoustics system [33]. In addition, the ANC problem in composite domains is analyzed in [28], [31], [32]. A series of physical experiments are discussed in [17], [18], [19] to validate the efficiency of this approach to ANC. One should note that the discussed technique is local since the operation of each control source is fully determined by the primary sound field nearby. This is a significant drawback of the approach because the primary field is not always available.

Instead of a discrete formulation, an alternative approach based on the continuous surface potentials is developed in [25], [26]. Then, the continuous control sources on the Huygens surface are approximated by the discrete distribution of control sources consisting of monopoles and dipoles. Since the Calderón potentials have a projection property, they provide an opportunity to retain a desired sound unaffected. Meanwhile, a significant issue with the approaches mentioned above is that they are only suitable for a fine enough grid to guarantee the projection property of the potentials. This might not be suitable for practical applications where a dense distribution of the control elements should be avoided. In addition, as shown in [39], with a sparse grid of control elements, the reverse effect of the control sources on the input data becomes significant. These problems can be effectively overcome with the nonlocal ANC [36], [37], [39]. In this approach based on the Calderón potentials, the operation of each secondary source is nonlocal since it takes into account the distribution of current sound field over the entire Huygens control surface. Thus, the nonlocal ANC allows a sparse grid of control elements to be used.

In the present paper, we consider the ANC problem for a finite long cylinder generating broadband noise. The nonlocal ANC approach [17], [18], [19] is employed for this purpose. The effect of the cylinder on Green's function and total noise attenuation is studied. We test what the minimal number of control sources per wavelength still provides noise attenuation. It is demonstrated that the relative level of noise attenuation is remarkably improved as the distance from the cylinder increases. Moreover, we demonstrate that the well-known Nyquist limitation for sampling can be relaxed if the acoustic field on the Huygens control surface is axisymmetric. The rest of the paper is organized as follows. Noise propagation from a finite long cylinder is elaborated in Section 2. In Section 3, the ANC algorithm in both continuous and discrete formulations is theoretically studied. Two Green's functions for the free field and for the unbounded region with the effect of a cylinder are introduced. Section 4 contains numerical experiments and their discussion. The Conclusion is summarized in the final section.

## 2 Noise generated by a finite cylinder

**Figure 1:**
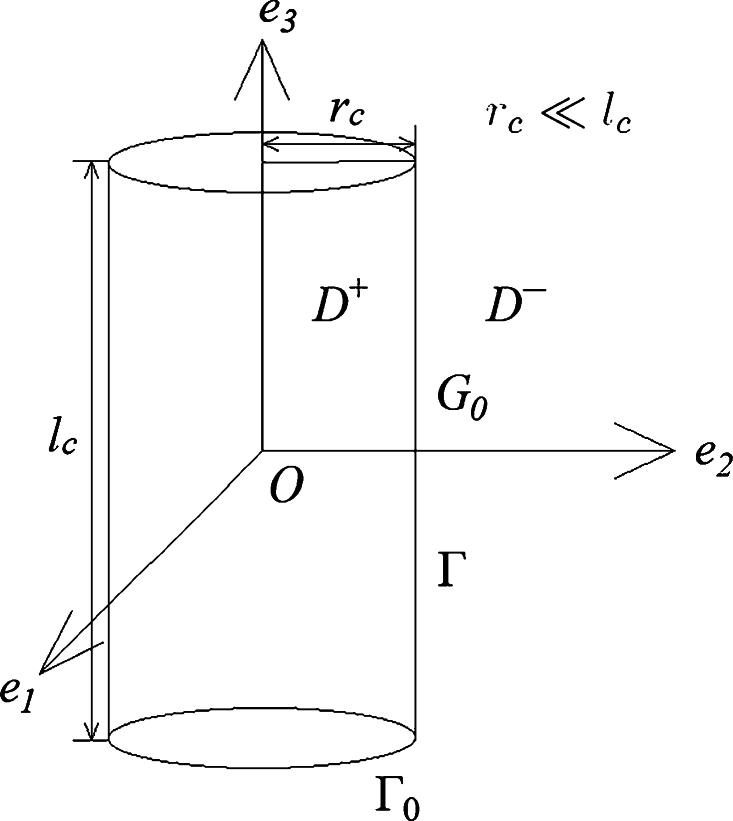
Sketch of a cylinder.

Consider a finite long cylinder $D^{+}$ of radius $r_{c}$ and length $l_{c}$. The aim of the ANC problem is to attenuate noise in the external space $D^{-}:=R^{3}﹨\bar{D^{+}}$. The entire boundary of the cylinder and its cylindrical side are denoted as $\Gamma_{0}$ and Γ, respectively: $\Gamma \subset \Gamma_{0}$ (see Fig. 1).

Suppose that $r_{c}\ll l_{c}$. In this case, to evaluate noise in the vicinity of the pipe, presume first that the pipe is infinite. In addition, presume that the noise distribution is uniform along the pipe. As such, we can neglect by the sound field nearby the ends of the pipe. Then, cylindrical sound waves are emitted due to the symmetry of the noise source.

Thus, the noise field $p_{0}(r)$ is described by the Helmholtz equation in the column coordinates:

(1) $$\frac{d^{2}p_{0}}{dr^{2}}(r)+\frac{1}{r}\frac{dp_{0}}{dr}(r)+k^{2}p_{0}(r)=0,$$

where *k* is the wave number. Here and everywhere further, under noise (sound) field we understand the Fourier-component corresponding to the appropriate wave number. The only variable *r* is the distance from the spatial point to the axis of symmetry.

The general solution of Eq. (1) is given by

(2) $$p_{0}(r)=A_{0}H_{0}^{\left(2\right)}(kr).$$

Here, $A_{0}$ represents the amplitude of the cylindrical sound wave, $H_{0}^{\left(2\right)}$ is the 0-th order Hankel function of the second kind.

Then, the noise generated by the section of the pipe is presented by the Green's formula:

(3) $$p(\text{x})=\int_{\Gamma_{0}}({p_{0}}_{|\Gamma_{0}}\frac{\partial G}{\partial \text{n}}-G{\frac{\partial p_{0}}{\partial \text{n}}}_{|\Gamma_{0}})d\sigma ,\text{x}\in D^{-}.$$

Here, *G* is the Green's function (see Appendix A), the sound pressure $p_{0}$ and its normal derivative $\partial p_{0}/\partial \text{n}$ are taken on the side of the finite cylinder. At any point on Γ, $p_{0}$ and $\partial p_{0}/\partial \text{n}$ are constant since $r=r_{c}$.

If $r_{c}\ll l_{c}$, then the closed surface integral in Eq. (3) can be approximated by the integral as follows, because the area of the top and bottom of the cylinder is relatively small compared to the whole surface area.

(4) $$p(\text{x})\approx p_{0}(r_{c})\int_{\Gamma }\frac{\partial G}{\partial \text{n}}d\sigma -\frac{\partial p_{0}}{\partial \text{n}}(r_{c})\int_{\Gamma }Gd\sigma ,\text{x}\in D^{-}.$$

Next, we can approximate the surface integrals in Eq. (4) introducing a set of discrete sources presented by monopoles and dipoles. Then, the noise field *p* generated by the entire lateral surface can be replaced by the total field from all discrete sources.

## 3 ANC algorithm

In this section, we initially give a brief description of the ANC problem. Then, the ANC algorithm based on the continuous surface potentials is provided to obtain the solution to the ANC problem. We introduce two Green's functions in the frequency domain. One of them corresponds to the free field, while the other takes into account the effect of the cylinder. The latter Green's function allows us to take into account the effect of the solid boundary of the pipe.

### 3.1 Description of the ANC problem

First, consider a boundary value problem (BVP) for the Helmholtz equation to describe the acoustic field in the frequency domain:

(5) $$(\Delta +k^{2})p=g,p\in \Xi .$$

Here, *g* represents the primary source generating the sound field *p*. Ξ is a function space such that the appropriate BVP is well-posed. In the case of the free space, Ξ contains functions satisfying the Sommerfeld boundary condition:

$$\lim_{r\to \infty }(r(\frac{\partial p}{\partial r}-ikp))=0.$$

To achieve volumetric noise attenuation in the ANC problem, the following inverse problem is solved with respect to the secondary source $G_{0}$, known as the control source:

(6) $$(\Delta +k^{2})p_{c}=g+G_{0},p_{c}\in \Xi ,\text{supp}\;G_{0}\subset \Gamma_{0}$$

such that

(7) $${p_{c}}_{D^{-}}=0,$$

where ${p_{c}}_{D^{-}}$ is the restriction of $p_{c}$ to the domain $D^{-}$.

This means that the noise should be eliminated in $D^{-}$ with the contribution of the secondary source $G_{0}$.

### 3.2 Solution of the ANC problem

If the primary field is available, then noise cancelation is immediately achieved in $D^{-}$ with $G_{0}$ [20], [35]:

(8) $$G_{0}={\frac{\partial p}{\partial \text{n}}}_{|\Gamma_{0}}\delta (\Gamma_{0})-p_{|\Gamma_{0}}\frac{\partial \delta (\Gamma_{0})}{\partial \text{n}}.$$

Here, **n** represents the inward normal vector to $\Gamma_{0}$, $\delta (\Gamma_{0})$ denotes the surface delta-function assigned to $\Gamma_{0}$. Thus, the control source $G_{0}$ is entirely determined by the sound pressure *p* and its normal derivative ∂p/∂n on $\Gamma_{0}$. Both *p* and ∂p/∂n. The control source $G_{0}$ represents the sum of single- and double-layer source terms, respectively.

With the implementation of the control source $G_{0}$, the secondary field $p_{g}$ to compensate for noise $p^{-}$ in $D^{-}$ can be derived thanks to Green's formula:

(9) $$p_{g}(\text{x})=-\int_{\Gamma_{0}}(p_{|\Gamma_{0}}\frac{\partial G}{\partial \text{n}}-G{\frac{\partial p}{\partial \text{n}}}_{|\Gamma_{0}})d\sigma =-p^{-}(\text{x}),\;\;\;\text{x}\in D^{-}.$$

Hence, the secondary field $p_{g}$ is the combination of single- and double-layer potentials.

It is to be noted that the surface integral in (9) represents the Calderón potential [20] in $D^{-}$:

(10) $$P_{D^{-}}\xi_{\Gamma_{0}}(\text{x})=\int_{\Gamma_{0}}(\frac{\partial G}{\partial \text{n}}(\text{x}|\text{y})\xi_{\Gamma_{0}|1}(\text{y})-G(\text{x}|\text{y})\xi_{\Gamma_{0}|2}(\text{y}))d\sigma ,$$

where $\text{x}\in D^{-}$, $\text{y}\in \Gamma_{0}$, with the density $\xi_{\Gamma_{0}}={\left(p,\frac{\partial p}{\partial \text{n}}\right)}_{|\Gamma_{0}}^{T}$.

Next, introduce the trace of an arbitrary smooth function w∈Ξ on $D^{-}$:

(11) $${\text{Tr}}_{\Gamma_{0}}\;w_{D^{-}}={\left(w,\frac{\partial w}{\partial \text{n}}\right)}_{|\Gamma_{0}}^{T}.$$

Here and further, $v_{D^{-}}$ means the restriction of the arbitrary function *v* to $D^{-}$.

Then, Eq. (9) represents the projection property of the potential:

(12) $$P_{D^{-}}{\text{Tr}}_{\Gamma_{0}}\;p_{D^{-}}=p_{D^{-}}^{-}.$$

An advantage of the Calderón potentials is that they do not presume the knowledge of Green's function. Instead, they can be either measured or computed using the method of difference potentials [29], [30] in advance.

### 3.3 Discretization of the density of surface potential

To implement the single- and double-layer control sources, they are substituted by monopoles and dipoles following [25]. For this purpose, the surface potential is further approximated by its discrete form.

As shown above, the surface integral on the cylindrical Huygens surface $\Gamma_{0}$ is approximated by the integral on Γ in Eq. (4). Similarly, if $r_{c}\ll l_{c}$, the approximation of surface potential in Eq. (9) is provided:

(13) $$p_{g}(\text{x})\approx -p_{0}(r_{c})\int_{\Gamma }\frac{\partial G}{\partial \text{n}}d\sigma +\frac{\partial p_{0}}{\partial \text{n}}(r_{c})\int_{\Gamma }Gd\sigma ,\;\;\;\text{x}\in D^{-}.$$

Correspondingly, the source $G_{0}$ is given by

(14) $$G_{0}\approx \frac{\partial p_{0}}{\partial \text{n}}(r_{c})\delta (\Gamma )-p_{0}(r_{c})\frac{\partial \delta (\Gamma )}{\partial \text{n}}.$$

The control source (14) formally provides attenuation of noise propagating from the surface Γ of the cylinder. The problem is that it is not realizable because it is continuously distributed over Γ. For practical realization, it should be approximated by a set of discrete sources.

Next, consider a uniform grid on the surface Γ. Each control source is placed onto one of the segments $\Delta \sigma_{i}\;\;(i=1,...,N_{c})$ where $N_{c}$ is the total number of non-intersecting segments covering the entire surface Γ. Thus, discrete approximations of Eqs. (13) and (14) are given by

(15) $$p_{g}(\text{x})\approx -\sum_{i=1}^{N_{c}}[p({\text{y}}_{i})\frac{\partial G}{\partial \text{n}}(\text{x}|{\text{y}}_{i})-G(\text{x}|{\text{y}}_{i})\frac{\partial p}{\partial \text{n}}({\text{y}}_{i})]\Delta \sigma_{i},$$

(16) $$G_{0}\approx \sum_{i=1}^{N_{c}}[\frac{\partial p}{\partial \text{n}}(r_{c})\delta (\text{y}-{\text{y}}_{i})-p(r_{c})\frac{\partial \delta }{\partial \text{n}}(\text{y}-{\text{y}}_{i})]\Delta \sigma_{i}.$$

Here, y∈Γ, ${\text{y}}_{i}$ denotes the vector to an i-th node for the control source on Γ. Thus, the single- and double-layer source terms are replaced by monopoles and dipoles in Eq. (16).

It is to be noted that the superposition of noise and secondary field presented by Eqs. (4) and (15), respectively, gives us residual noise after ANC.

### 3.4 Green's function

The surface potential can be explicitly approximated if Green's function is available.

The free field Green's function $G_{f}$ is given by

(17) $$G_{f}=\frac{e^{ikr_{s}}}{4\pi r_{s}}.$$

Here, $r_{s}$ is the distance between a point source and an observation point. This Green's function does not take into account the effect of the solid pipe.

In the case of a solid cylinder, presume that the homogeneous Neumann boundary condition is satisfied on Γ. Then, an approximate expression of Green's function can be found thanks to the stationary phase theorem: [5]:

(18) $$G_{c}=\frac{e^{ikr^{\prime }}}{4\pi r^{\prime }}[J_{0}(\frac{kr_{x}r_{c}}{r^{\prime }})-\alpha_{0}(\frac{kr_{x}r_{c}}{r^{\prime }})H_{0}^{\left(1\right)}(\frac{kr_{x}r_{c}}{r^{\prime }})].$$

Here, $J_{0}$ and $H_{0}^{\left(1\right)}$ denote the first kind of 0-th Bessel function and the first kind of 0-th Hankel function, respectively; $\alpha_{0}(u)={J_{0}}^{\prime }(u)/{H_{0}^{\left(1\right)}}^{\prime }(u)$; $r_{x}$ is the distance from the observation point to the axis of symmetry of the cylinder; $r^{\prime }=|\text{x}-y_{3}{\text{e}}_{3}|$, where **x** is the vector to the observation point, y∈Γ, $y_{3}{\text{e}}_{3}$ is the component of **y** along the axis of symmetry. As can be seen, $r^{\prime }$ corresponds to the distance from a source to the receiver where the radius of the cylinder is neglected.

In the next section, we employ these Green's functions, $G_{f}$ and $G_{c}$, in numerical experiments.

## 4 Numerical experiments

### 4.1 Experimental setup

**Figure 2:**
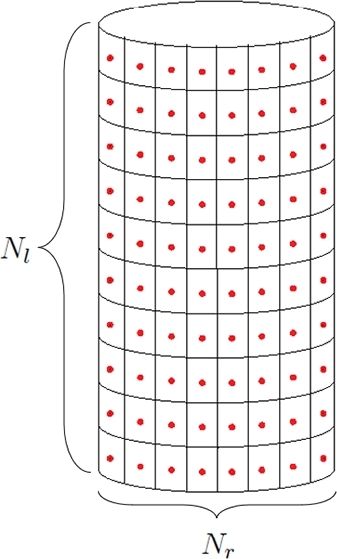
Sketch of distribution of control sources over the pipe surface.

**Figure 3:**
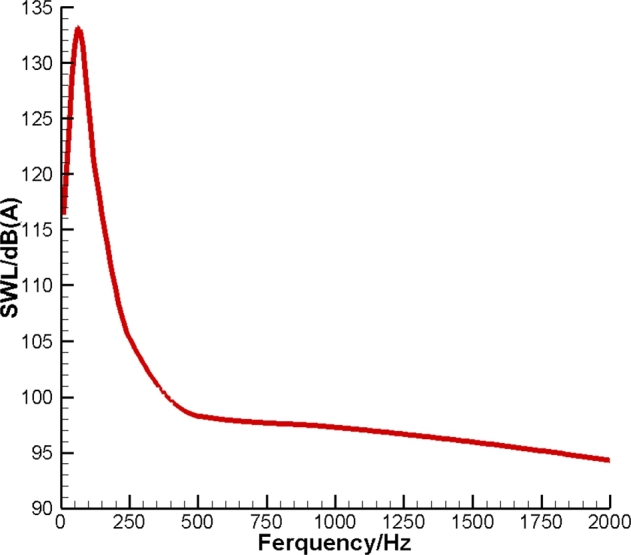
Sound power level generated by the pipe.

In this section, we consider numerical experiments based on the broadband sound signal to test the efficiency of the ANC technique for a pipe. First, consider a finite pipe with the length $l_{c}$ of 100m and radius $r_{c}$ of 2m in an unbounded domain. For the sake of simplicity, we neglect the effect of the ground. The control sources are uniformly assigned on the surface of the pipe as shown in Fig. 2. The pipe is equally divided into $N_{t}$ segments. There are $N_{l}$ annular layers of control sources along the axis of symmetry. In each layer, $N_{r}$ control sources are located at the geometric center of each segment. Thus, we have $N_{t}=N_{l}\cdot N_{r}$ control sources in total. The area of each segment is equal to $\Delta \sigma =2\pi r_{c}l_{c}/N_{t}$. Each control source is supposed to be a combination of a monopole and dipole. In turn, assume that the noise generated from the pipe is uniformly distributed along its length. We presume that the sound power level (SWL) of the original noise follows the distribution shown in Fig. 3. This distribution of noise can be grouped into seven octave bands as shown in Table 1. Noise above 2kHz is neglected as it rapidly declines with the distance from the pipe.

**Table 1:** Octave bands of noise generated by the pipe.

Octave band	*f* [Hz]	A	SWL [dB(A)]	SWL [dB]
0	31.5	-39.4	125.1	164.5
1	63	-26.2	133.3	159.5
2	125	-16.1	120.3	136.4
3	250	-8.6	105.3	113.9
4	500	-3.2	98.3	101.5
5	1000	0	97.3	97.3
6	2000	1.2	94.3	93.1

To investigate the efficiency of ANC with the variation of distance, we consider the noise attenuation up to 2km away from the pipe along a line orthogonal to the axis of the cylinder and situated in the plane of symmetry.

Before the discussion of the numerical results, it should be noted that SWL only depends on the acoustic power generated by the sound source. Therefore, we only use SWL to determine the power of sound emitted by the pipe in each octave band. However, in contrast to SWL, the sound pressure level (SPL) is directly related to the distance from the acoustic source. Thus, SPL allows us to express the amplitude of sound at each observation point. Both SWL and SPL can be expressed with the units dB and dB(A). SPL in dB yields: $\mathrm{SPL}[\mathrm{dB}]=10\mathrm{lg}(p^{2}/p_{ref}^{2})$, where $p_{ref}=2\cdot 10^{-5}$ Pa. We use MATLAB to carry out numerical simulations based on the appropriate Green's function.

### 4.2 Numerical results and discussion

First, consider the noise attenuation with the variation of distance for different frequencies grouped in the octaves. It is clear that both original (not affected by ANC) and residual (affected by ANC) noise decline with the distance from the cylinder. It turns out the relative residual noise (scaled by the original noise) also declines. This effect is analytically explained in Appendix B.

**Figure 4:**
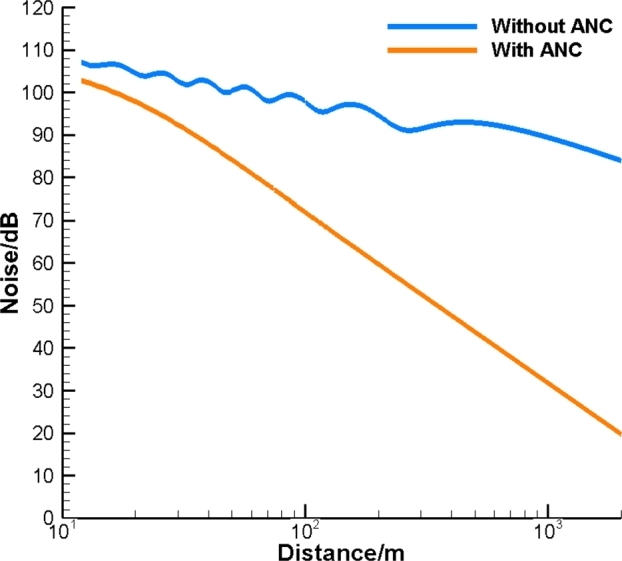
Noise attenuation in the first-octave band with *G*_*c*_, (*N*_*r*_,*N*_*l*_)=(11,20).

To attenuate noise in the first-octave band, consider the distribution of control sources on the solid cylindrical surface to be $\left(N_{r},N_{l})=(11,20\right)$. The Green's function $G_{c}$ is used first. As shown in Fig. 4, the gap between the noise with and without ANC is less than 5dB at 10m. Then, along with the growth of distance, the gap is remarkably enlarged to 26dB at 100m and 57dB at 1km, respectively. Finally, even 64dB noise attenuation is provided by ANC at 2km away from the pipe. At this distance, the noise field without ANC is over 80dB while the level of noise with ANC is less than 20dB. Therefore, it is demonstrated that the relative noise attenuation drastically increases with the distance. Compared to the noise without ANC, the residual noise after ANC remarkably drops as distances from the cylinder increases. It is to be noted that the results of noise attenuation are almost identical to the case with the free-field Green's function $G_{f}$. Thus, the effect of the solid cylinder can be ignored if a fine enough grid of control sources is applied.

**Figure 5:**
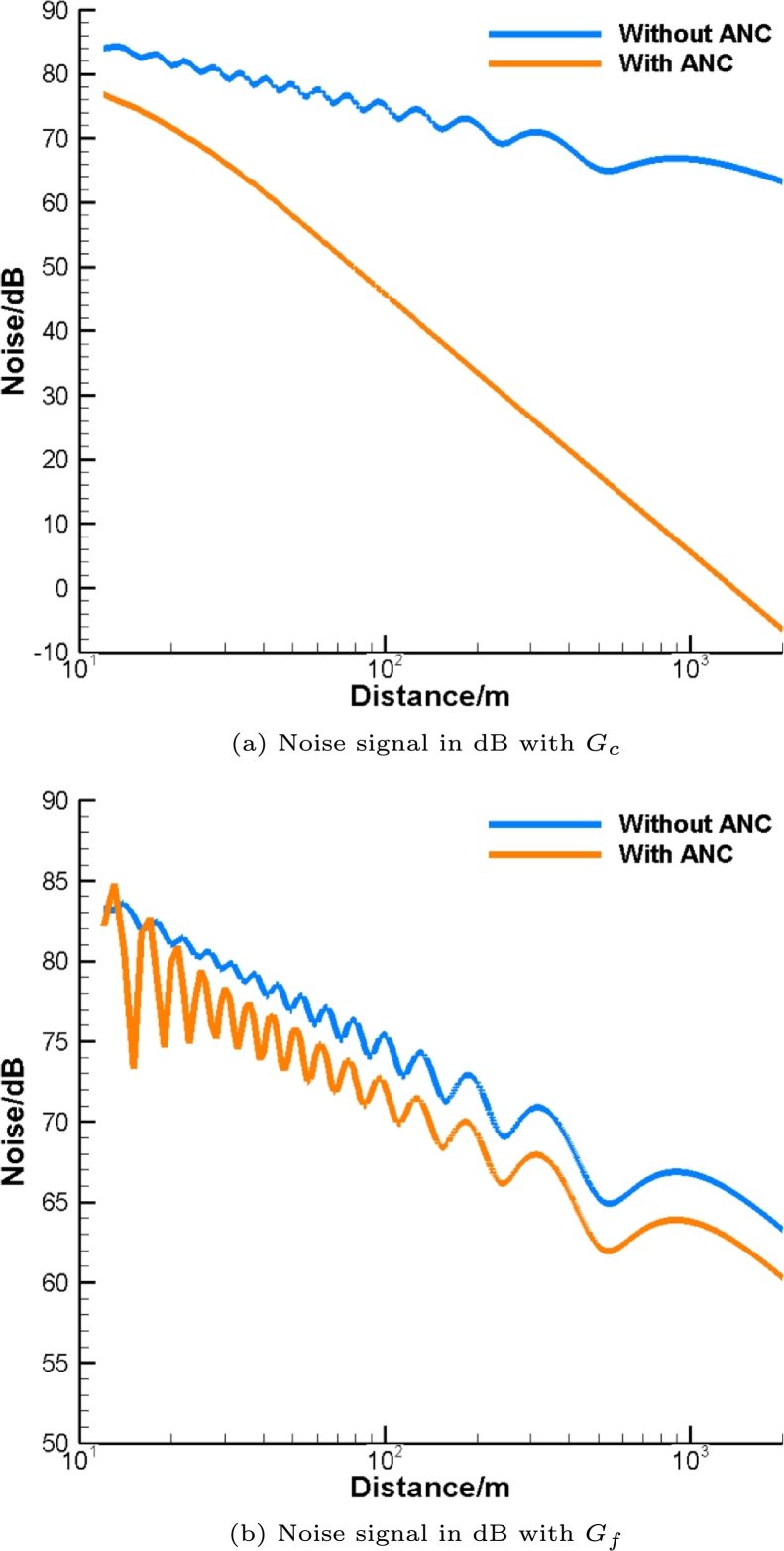
Noise attenuation in the second-octave band with (*N*_*r*_,*N*_*l*_)=(6,40).

Next, we consider the control sources on a relatively sparse grid for relatively high frequencies. For this purpose, we use a coarse distribution $\left(N_{r},N_{l})=(6,40\right)$ with the second-octave band. In this case, there are less than two control sources per wavelength for both annular and axial directions. As can be seen in Fig. 5(a), noise with ANC falls rapidly compared to the case without active control. It is to be noted that if the free-field Green's function $G_{f}$ (see Fig. 5(b)), only a small gap about 3 dB between curves is retained at long distances. From the comparison of the results in Fig. 5(a) and Fig. 5(b), it is clear that ANC with $G_{c}$ provides much better noise attenuation than that with $G_{f}$. In other words, the presence of solid cylinder improves the efficiency of the ANC technique. Numerical experiments show that this improvement only occurs when the number of control sources per wavelength for both two directions is in the range between one and two.

**Figure 6:**
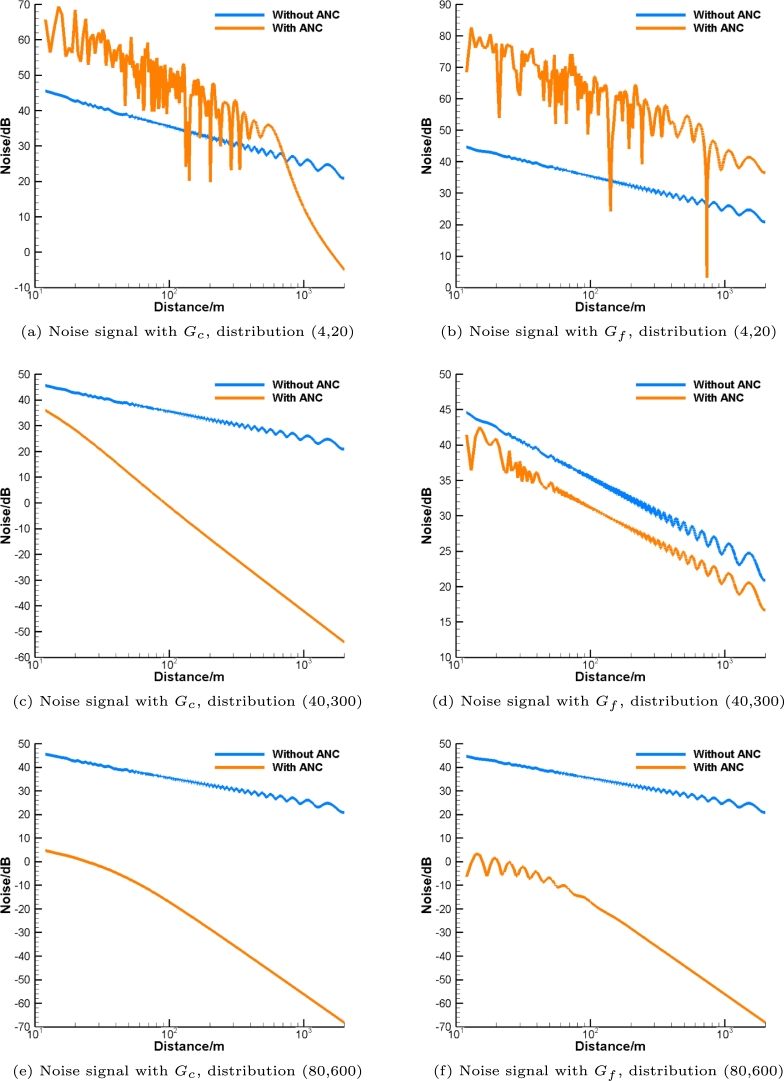
Noise attenuation in the fifth-octave band.

The next numerical experiments are carried out to test the minimal number of control sources per wavelength required for essential noise attenuation. Consider noise emitted in the fifth-octave band. First, presume that $\left(N_{r},N_{l})=(4,20\right)$. As can be seen in Fig. 6(b), noise is drastically reinforced over the whole distance as the control nodes are distributed too sparsely. As shown in Fig. 6(a), the noise is amplified when the distance is less than 700m while it diminishes at a longer distance.

If $\left(N_{r},N_{l})=(40,300\right)$, then slightly more than one control source per wavelength in both directions is present. Similar to the results in Fig. 5, noise significantly drops when the distance increases as shown in Fig. 6(c). At first glance this result is counterintuitive because it contradicts the Nyquist-Shannon sampling theorem since less than two control sources per wavelength are used. This effect can be explained as follows. The sound pressure and its normal derivative are constant on the surface of the cylinder because of the symmetry. As shown in Appendix B, in this case the residual noise should vanish as $1/r^{2}$ rather than 1/r, where *r* is the distance to the cylinder. In addition, the resolution of the waves on the surface is not needed because the acoustic field is uniform on the entire surface thanks to the symmetry. Thus, much fewer control sources are required for ANC. Finally, it is worth also noting that with the free-field Green's function, only about 4dB of noise attenuation is achieved as can be seen in Fig. 6(d). Thus, the effect of the solid surface of the pipe is significant.

In the next experiment, we double both $N_{c}$ and $N_{l}$ to 80 and 600. As shown in Figs. 6(e) and 6(f), noise attenuation by ANC with $G_{c}$ and $G_{f}$ is remarkably almost the same. This is because there are more than two control sources per wavelength. The denser grid for the control sources appreciably boosts the level of noise attenuation.

Thus, noise is not reduced but reinforced when the number of control sources per wavelength is less than one. Satisfactory noise attenuation is provided when the number of control sources per wavelength is in the interval between one and two. If the number of control sources per wavelength is greater than two, noise is significantly suppressed, and the results of ANC with $G_{c}$ and $G_{f}$ are identical. This means that the effect of the cylinder wall on noise attenuation vanishes.

**Figure 7:**
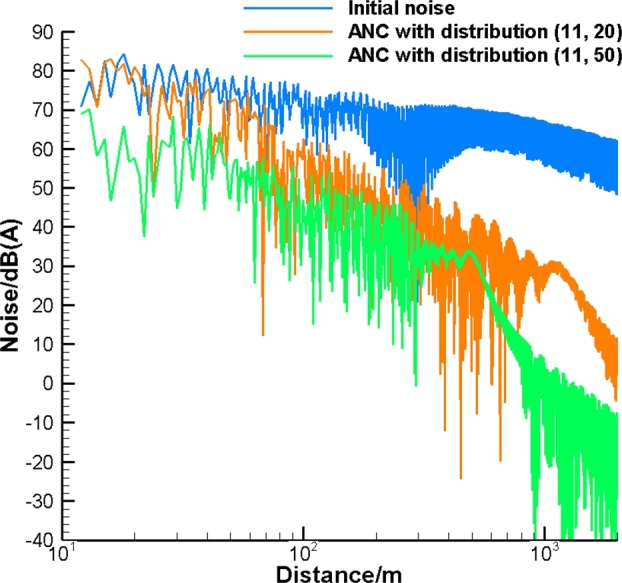
Broadband noise attenuation by ANC with *G*_*c*_.

**Figure 8:**
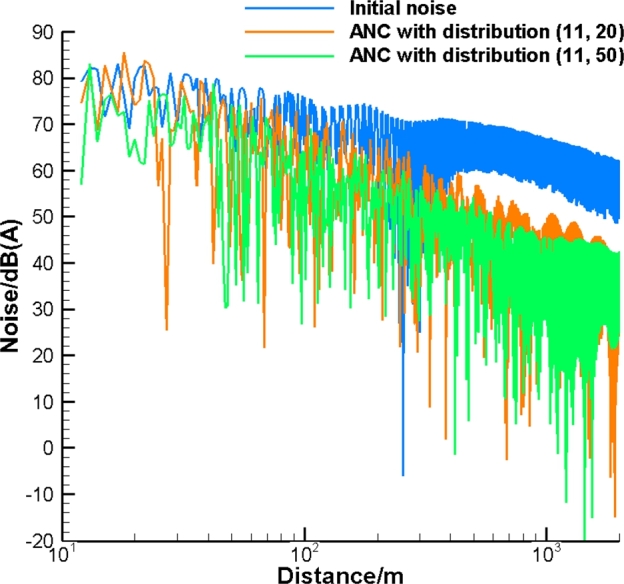
Broadband noise attenuation by ANC with *G*_*f*_.

**Figure 9:**
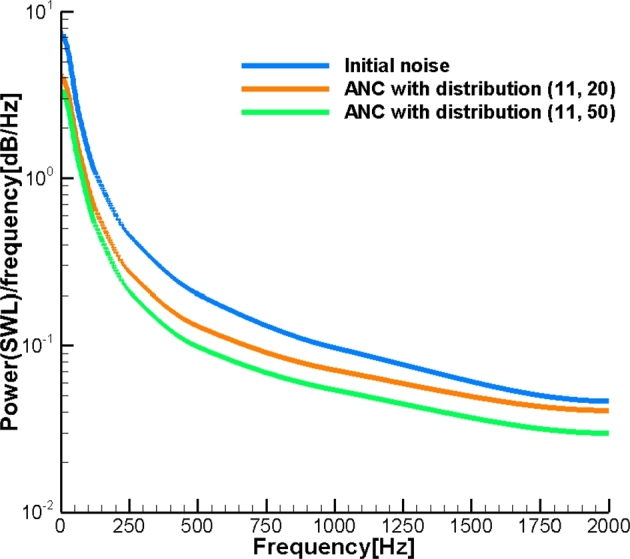
Power spectral density by ANC with *G*_*c*_.

**Figure 10:**
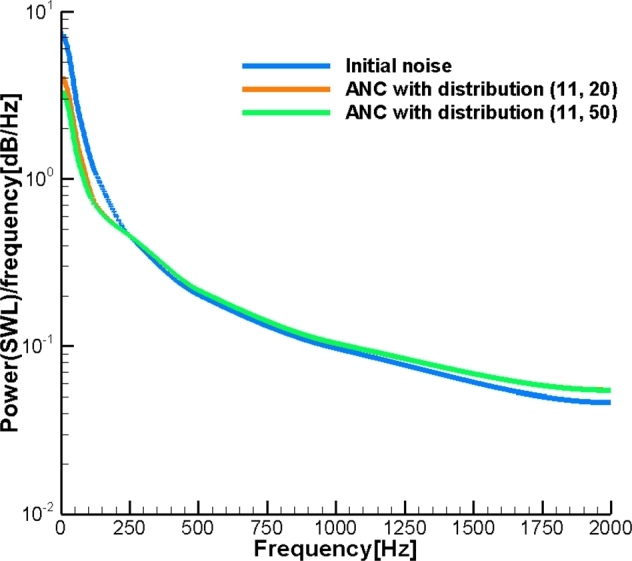
Power spectral density by ANC with *G*_*f*_.

Finally, presume noise emitted by the pipe comprises the entire range of frequency with SWL corresponding to Fig. 3. This model simulates the noise cancelation process for an industrial pipeline generating multi-frequency noise. This allows us to assess the feasibility of the introduced ANC system with a relatively small number of control sources for industrial applications. The number of control sources corresponds to $N_{r}=11$ while $N_{l}$ is equal to 20 and 50. To assess the effect of noise on the human ear, we use dB(A). In Fig. 7 the results are based on the Green's function $G_{c}$ with the effect of the cylinder, while in Fig. 8 the results are obtained with the free field Green's function $G_{f}$. As can be seen, the broadband noise is effectively attenuated in the far field. The more compact distribution of the control sources provides a higher level of noise attenuation. In addition, the effect of the cylinder improves noise attenuation. This occurs because of the coarse grid of control sources used. The corresponding power spectral density (PSD) for ANC with $G_{c}$ and $G_{f}$ is illustrated in Figure 9, Figure 10, respectively. In Fig. 10, ANC with $G_{f}$ attenuates noise at low frequencies while amplifying noise at high frequencies. Compared to the results of ANC with $G_{c}$ and $G_{f}$, it can be found that the existence of the solid cylinder improves noise attenuation in the range of high frequencies.

As noted above, the assumptions of the axisymmetric original noise field and its uniform distribution along the pipe are important for significantly reducing the number of active controls. It may be violated in real-life applications. However, the noise field can be split into a mean field satisfying these assumptions and a residual field. The residual noise is not necessarily attenuated with the minimal number of controls required for the mean field. However, if the level of the residual noise is small, this might be acceptable. In the analysis, we also consider the controls as point sources. For the validity of this model, the minimal wavelength should be much greater than the real size of each control source.

## 5 Conclusion

The active noise control problem for a finite cylinder emitting broadband noise has been studied. The effect of the cylinder on the Green's function and the total noise reduction was considered. It was shown that a significant level of noise attenuation can be achieved with a relatively small number of control sources.

It has been found that the effect of noise attenuation remarkably improves with increasing the distance from the pipe if the number of control sources per wavelength is sufficient. In some cases, the noise can be amplified in the near field and then reduced in the far field. This effect occurs because the error of discrete approximation of the surface potential for the control source drastically declines with the growth of distance. It can allow us to configure the number of control sources according to the distance of the protected object from the pipe. In addition, it turns out that the effect of the cylinder improves the performance of the ANC algorithm when the control sources are sparsely distributed. Finally, it has been discovered that even less than two control sources per wavelength along each direction can provide essential noise attenuation. This result overcomes the Nyquist limitation thanks to the uniform acoustic field on the Huygens surface. In addition, it turns out that in this case the effect of the solid surface is significant. Otherwise, if the distribution of control sources is dense enough, the effect of the solid surface can be neglected.

## CRediT authorship contribution statement

**N. Hu:** Writing – original draft, Visualization, Validation, Software, Investigation. **S. Utyuzhnikov:** Writing – review & editing, Supervision, Methodology, Investigation, Formal analysis, Conceptualization.

## Declaration of Competing Interest

The authors declare the following financial interests/personal relationships which may be considered as potential competing interests: Nan Hu reports financial support was provided by 10.13039/501100000770The University of Manchester. Sergey Utyuzhnikov reports a relationship with The University of Manchester that includes: employment. If there are other authors, they declare that they have no known competing financial interests or personal relationships that could have appeared to influence the work reported in this paper.

## Appendix A Introduction to the frees-pace Green's function

The free-space Green's function G(x|y) in the frequency domain is the casual solution of the Helmholtz equation generated by the point source δ(x−y) located at **y**:

(A.1) $$(\Delta +k^{2})G(\text{x}|\text{y})=\delta (\text{x}-\text{y}).$$

Thus,

$$G(\text{x}|\text{y})=\frac{e^{ik|\text{x}-\text{y}|}}{4\pi |\text{x}-\text{y}|}.$$

## Appendix B Error evaluation for surface potentials

Consider the error of the approximation of the continuous single-layer potential.

(B.1) $$\epsilon_{m}=\int_{\Gamma }{\frac{\partial p}{\partial \text{n}}}_{|\Gamma }(\text{y})G(\text{x}|\text{y})d\sigma -\sum_{i=1}^{N_{c}}\frac{\partial p}{\partial \text{n}}({\text{y}}_{i})G(\text{x}|{\text{y}}_{i})\Delta \sigma_{i}=\sum_{i=1}^{N_{c}}\int_{\Gamma_{i}}[{\frac{\partial p}{\partial \text{n}}}_{|\Gamma }(\text{y})-\frac{\partial p}{\partial \text{n}}({\text{y}}_{i})]G(\text{x}|\text{y})d\sigma +\sum_{i=1}^{N_{c}}\frac{\partial p}{\partial \text{n}}({\text{y}}_{i})\int_{\Gamma_{i}}[G(\text{x}|\text{y})-G(\text{x}|{\text{y}}_{i})]\Delta \sigma_{i}.$$

Here, **x** is the vector to the observation point, ${\text{y}}_{i}$ is the vector to an i-th control source.

Consider the far-field condition, $\sqrt{\Delta \sigma_{i}}\ll r_{i}=|\text{x}-{\text{y}}_{i}|$. Following the Taylor expansion, we have

$$G(\text{x}|\text{y})-G(\text{x}|{\text{y}}_{i})∼\frac{1}{\left|\text{x}-\text{y}\right|}-\frac{1}{r_{i}}\approx \frac{\text{x}\cdot \Delta \text{y}}{{r_{e}}^{3}},$$

where $\text{y}\in \Gamma_{i}$, $r_{i}=|\text{x}-{\text{y}}_{i}|\approx r_{e}=|\text{x}-\text{y}|$, $\Delta \text{y}=\text{y}-{\text{y}}_{i}$.

Thus,

$$\int_{\Gamma_{i}}[G(\text{x}|\text{y})-G(\text{x}|{\text{y}}_{i})]\Delta \sigma_{i}∼\frac{{\left(\sqrt{\Delta \sigma_{i}}\right)}^{3/2}}{{r_{e}}^{2}}.$$

On the other hand,

$${\frac{\partial p}{\partial \text{n}}}_{|\Gamma }(\text{y})-\frac{\partial p}{\partial \text{n}}({\text{y}}_{i})∼\frac{\partial }{\partial s}\frac{\partial p}{\partial \text{n}}({\text{y}}_{i})\sqrt{\Delta \sigma_{i}}.$$

Thus,

(B.2) $$\int_{\Gamma_{i}}[{\frac{\partial p}{\partial \text{n}}}_{|\Gamma }(\text{y})-\frac{\partial p}{\partial \text{n}}({\text{y}}_{i})]G(\text{x}|\text{y})d\sigma ∼\frac{\partial }{\partial s}\frac{\partial p}{\partial \text{n}}({\text{y}}_{i})\frac{{\left(\Delta \sigma_{i}\right)}^{3/2}}{r_{e}},$$

while

$$\frac{\partial p}{\partial \text{n}}({\text{y}}_{i})\int_{\Gamma_{i}}[G(\text{x}|\text{y})-G(\text{x}|{\text{y}}_{i})]\Delta \sigma_{i}∼\frac{\partial p}{\partial \text{n}}({\text{y}}_{i})\frac{{\left(\Delta \sigma_{i}\right)}^{3/2}}{{r_{e}}^{2}}.$$

Here, ∂/∂s is the derivative along the surface. It is proportional to 1/λ, where *λ* is the wave length.

Thus, if $r_{e}\gg \lambda$, then the last term in (B.1), which is related to the local variation of Green's function, is usually much less than the first term.

However, if the acoustic field is the same on the surface Γ, the first term in (B.1) is equal to zero. Thus, the last term becomes the leading term in the error in noise attenuation. Then, the local error for i-th segment is

$$\epsilon_{i}∼\frac{{\left(\Delta \sigma_{i}\right)}^{3/2}}{{r_{e}}^{2}}.$$

Consider the summation of $\epsilon_{i}$ on the whole surface. The total error is

$$\epsilon_{m}∼\frac{\sqrt{\Delta \sigma_{i}}S}{{r_{e}}^{2}}∼\frac{\lambda S}{{r_{e}}^{2}},$$

while the noise is proportional to $S/r_{e}$. Here, *S* represents the area of the surface.

Thus, if the typical size between the nodes is about *λ*, then in distances $\lambda \ll r_{e}$ after ANC noise should vanish $\lambda /r_{e}$ faster than that without ANC. It is not the case if the acoustic field varies on the surface Γ. In this case the contribution of the first term in (B.1) determines the behavior of the error that would vanish comparably with the original noise.

The contribution of the dipoles is similar: there is the leading term that is proportional to the monopole field divided by *λ*.

Thus, the total error of approximation for the single- and double-layer potentials decreases with the increase of distance $r_{e}$. This explains why higher level of noise attenuation is achieved with the growth of distance $r_{e}$.
